# Machine Learning-based Classification for the Prioritization of Potentially Hazardous Chemicals with Structural Alerts in Nontarget Screening

**DOI:** 10.1021/acs.est.4c10498

**Published:** 2025-03-07

**Authors:** Nienke Meekel, Anneli Kruve, Marja H. Lamoree, Frederic M. Been

**Affiliations:** † KWR Water Research Institute, P.O. Box 1072, Nieuwegein 3430 BB, The Netherlands; ‡ Chemistry for Environment and Health, Amsterdam Institute for Life and Environment (A-LIFE), Vrije Universiteit, De Boelelaan 1085, Amsterdam 1081 HV, The Netherlands; § Department of Materials and Environmental Chemistry, Stockholm University, Stockholm SE-106 91, Sweden; ∥ Department of Environmental Science, Stockholm University, Stockholm SE-106 91, Sweden

**Keywords:** nontarget screening, structural alerts, machine learning, prioritization, toxicity, mass spectrometry

## Abstract

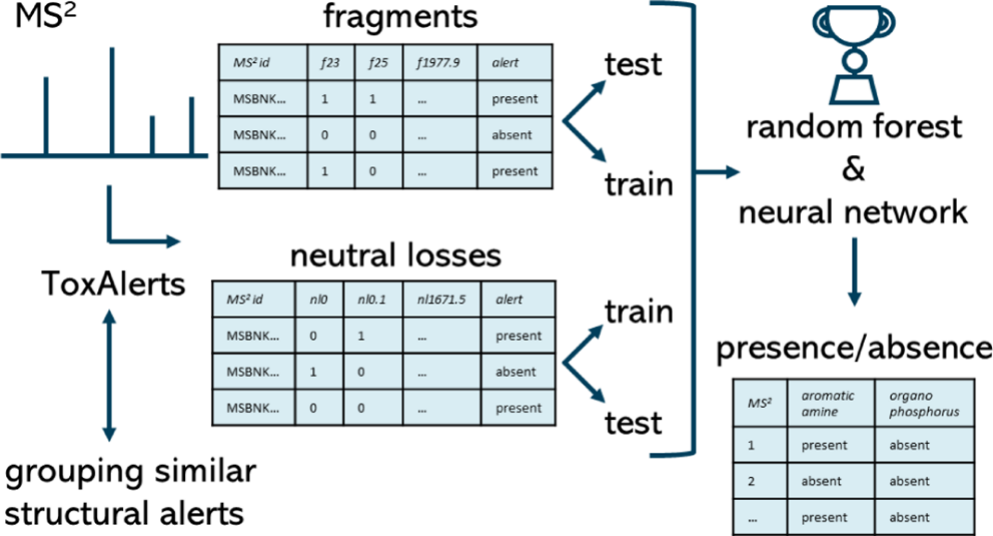

Nontarget screening (NTS) with liquid chromatography high-resolution mass spectrometry (LC-HRMS) is commonly used to detect unknown organic micropollutants in the environment. One of the main challenges in NTS is the prioritization of relevant LC-HRMS features. A novel prioritization strategy based on structural alerts to select NTS features that correspond to potentially hazardous chemicals is presented here. This strategy leverages raw tandem mass spectra (MS^2^) and machine learning models to predict the probability that NTS features correspond to chemicals with structural alerts. The models were trained on fragments and neutral losses from the experimental MS^2^ data. The feasibility of this approach is evaluated for two groups: aromatic amines and organophosphorus structural alerts. The neural network classification model for organophosphorus structural alerts achieved an Area Under the Curve of the Receiver Operating Characteristics (AUC-ROC) of 0.97 and a true positive rate of 0.65 on the test set. The random forest model for the classification of aromatic amines achieved an AUC-ROC value of 0.82 and a true positive rate of 0.58 on the test set. The models were successfully applied to prioritize LC-HRMS features in surface water samples, showcasing the high potential to develop and implement this approach further.

## Abstract 2

This study reports on the development and application of a machine learning tool to predict the presence of potentially hazardous features from raw tandem mass spectra.

## Introduction

Drinking water sources globally are increasingly under pressure due to drought, salinization, and contamination by chemicals, etc., where part of the chemical contamination is caused by organic micropollutants.^1^ This umbrella term covers a wide variety of substances present at trace levels, i.e., μg/L range or lower, and originating from a wide range of anthropogenic activities.^2^ Some of these organic micropollutants are monitored intensively using liquid chromatography coupled to high-resolution mass spectrometry (LC-(HR)MS). Yet many chemical contaminants and their transformation products are still unknown.^3,4^ Nontarget screening (NTS), often combined with suspect screening, is increasingly used to detect these unknown chemicals.^5,6^ NTS of surface water samples, which can be regarded as relatively simple compared to other matrices like soil or blood, often results in the detection of up to a few thousand LC-HRMS features.^7^ It is extremely laborious to identify all of them, and a substantial proportion is probably naturally occurring, so only the most relevant LC-HRMS features should be prioritized for further investigation or identification. Commonly used prioritization strategies leverage feature intensity, occurrence, trends/pattern analysis, removal rate, transformation products, source, usage data, and available metadata.^8−11^ Recent studies^12,13^ have shown that toxicity is crucial for prioritization, given that this is one of the main aspects of interest in environmental screening. Prioritization on toxicity can be done on either suspect candidates (i.e., structures), for example,^12,14^ or on unknown features. The latter is more challenging since there is no information available on the chemical structure of the feature. To address toxic effects and hazards in the prioritization of unknown features, several *in vitro* and *in silico* tools have been developed lately. Examples of these tools are *in vitro* effect-directed analysis^15,16^ or risk-based using available (semi)-quantitative and toxicity information.^17^ Furthermore, *in silico* tools to aid the prioritization of hazardous substances have recently been developed. For instance, MS2Tox^18^ and MLinvitroTox^19^ machine learning tools use SIRIUS + CSI:FingerID fingerprints^20^ of tandem mass spectra (MS^2^) to predict fish lethal concentration 50% (LC_50_) values for an unknown substance and toxicity values (active/not active) on selected bioassay endpoints, respectively. MS2Quant^21^ uses the same fingerprints to predict ionization efficiency values that can be used to estimate the concentration of the detected substances. These studies have shown that MS^2^ spectra can be used to obtain valuable information for risk-based prioritization of chemical features detected during monitoring with NTS. However, intermediate steps such as autoencoders^22,23^ or fingerprint prediction tools are required to describe MS^2^ spectra. Commonly applied techniques to encode information into descriptors based on MS^2^ spectra make use of fragmentation trees, machine learning techniques such as latent Dirichlet allocation,^24^ CLERMS,^25^ or a combination of both like in CSI:FingerID,^26^ where fragmentation trees and multiple kernel learning are combined.^27^ Arguably, predicted fingerprints or molecular descriptors might lead to information loss and increased uncertainty due to error propagation.^28^

Furthermore, an alternative approach to prioritize LC-HRMS features belonging to potentially toxic chemicals involves the use of so-called structural alerts. Also known as toxicophores, these are molecular substructures that are related to the toxic effects of a molecule. In a previous study,^29^ we demonstrated that some fragments and neutral losses in MS^2^ spectra can indicate the presence of a structural alert in the corresponding substance. A similar approach was used in another study by Lo Piparo et al.,^30^ where they found characteristic fragments in MS^2^ spectra of substances with a pyrrolizidine alkaloid structural alert that has been associated with genotoxicity. Similarly, Meng et al.^31^ found characteristic fragments for organophosphate esters in MS^2^ spectra of atmospheric pressure chemical ionization (APCI) and used these to detect novel organophosphate ester substances using NTS. Mayer et al.^32^ derived diagnostic fragments of trichothecenes in their ESI MS^2^ spectra, and Pu et al.^33^ used diagnostic fragments and neutral losses in experimental ESI MS^2^ spectra to study *N*-nitrosamines. The above-described approaches take advantage of the fact that similar molecules or molecules with similar functional groups might show similarities in MS^2^ spectra.^34^ This principle is also used in tools like MS2Query,^35^ which is used for analog search. In particular, fragments and neutral losses are commonly used for the prediction of structural characteristics from MS^2^ spectra^24,36^ and can be used to assess structural similarity.^37^

Here, we present a novel offline prioritization strategy using HRMS data that relies on the concept of structural alerts and is based on raw MS^2^ spectra without the use of fingerprints or autoencoders. This study explored whether the presence of various drinking water-relevant hazardous substances could be predicted based on the experimental MS^2^ of LC-HRMS features detected in NTS. We used fragment masses, hereafter referred to as “fragments”, and neutral losses to explore their predictive power for the presence of structural alerts. Two classifier models were developed to predict the presence of aromatic amine and organophosphorus structural alerts based on the composition of the MS^2^ spectrum. Last but not least, the approach is tested on environmental surface water samples.

## Materials and Methods

For model development, all data preprocessing, machine learning, and validation were conducted using R^38^ version 4.2.1, RStudio^39^ and the caret package.^40^ Calculations were performed on an HP Z6 G4 workstation with two Intel Xeon Gold 6134 CPUs at 3.20 GHz. For the application to NTS data, all data analysis was performed using R^38^ version 4.3.3 and the patRoon^41^ package on an HP ProBook with one Intel Core i7-8565U CPU @ 1.80 GHz. Visualization was done using the ggplot2 package.^42^

### Structural Alerts

The online web server ToxAlerts^43^ was used for the selection of structural alerts related to toxicity endpoints previously selected for their relevance to drinking water,^29^ i.e., endocrine disruption (*n* = 35, i.e., the number of structural alerts for this endpoint), developmental and mitochondrial toxicity (*n* = 12), nongenotoxic carcinogenicity (*n* = 23), and genotoxic carcinogenicity and mutagenicity (*n* = 117). The 187 alerts were examined manually, and related alerts were aggregated based on expert knowledge, using similarities in their carbon skeleton, heteroatoms, general structure, and functional groups and their positions relative to each other. This resulted in 32 groups (Table S1) and a remaining set of ungrouped alerts (Table S2). Two structural alert groups were selected for the development of the approach. The rationale for choosing these two alert groups is presented later in the results section. The first group, made up of 10 of the 187 initial structural alerts, was the aromatic amine, associated with genotoxic carcinogenicity and mutagenicity. The second group, consisting of 2 structural alerts, was the organophosphorus alert, associated with endocrine disruption.

### Data Set

MassBank Europe^44,45^ was used as a dataset for the model training. The dataset (90,398 unique mass spectra of 19,712 unique SMILES) was filtered for MS^2^ spectra recorded with electrospray ionization mass spectrometry, identification level 1, and available SMILES identifiers.^46^ This resulted in 7334 unique SMILES, which were screened for the presence of all 187 structural alerts. To this extent, the online tool “ToxAlerts”^43^ was used (v. 4.3.327). All spectra were labeled with “none” in case no alert was present or with the alert code(s) representing the alert(s) present in the corresponding molecule. Only spectra obtained with HRMS (i.e., with instrument code ESI-QTOF or ESI-ITFT) in positive ionization mode and obtained from adducts [M + H]^+^ were selected for further analysis. To simplify model training and development, we set the focus on [M + H]^+^ adducts. The number of unique substances and MS^2^ spectra per alert group is shown in Tables S1 and S2. For the model development, only the aromatic amine and organophosphorus alert groups were considered. An individual and tailored binary classification model was trained for each of them.

### Preprocessing

Fragment *m*/*z* or neutral losses computed from the spectra were used as input variables for model training. Neutral losses were calculated by subtracting each fragment with a relative intensity >50 (out of 999) from the precursor ion *m*/*z*. Only neutral losses >0 *m*/*z* were retained, as neutral losses <0 *m*/*z* were caused by fragments with larger masses than the precursor ions. Fragments with a relative intensity >50 and an *m*/*z* value below the precursor ion *m*/*z* were kept. All neutral losses and fragments were binned by rounding to the nearest tenth, i.e., 0.1 *m*/*z*. Spectra without neutral losses and fragments were removed from the data sets. The data sets were arranged in two binary matrices: one matrix with 3531 unique fragments (ranging from 26 *m*/*z* up to 1229.6 *m*/*z*) and another matrix with 4408 unique neutral losses (ranging from 0 *m*/*z* up to 1155.1 *m*/*z*), both corresponding to the same 23,387 unique spectra. Every instance (row) represented a spectrum, and every feature (column) represented a fragment or neutral loss. The presence of a fragment or neutral loss in the spectrum was coded as ‘1′, while absence was coded as “0”.

Specific subsets of spectra were made for the two alert groups, containing spectra with the alert and a random sample of 7600 MS^2^ spectra without the alert of interest. Fragments or neutral losses that were absent in all of these spectra in the subset were removed from the data set. Some fragments or neutral losses were always occurring together; therefore, these were removed to avoid having correlated predictors in the data set. An additional preprocessing step was applied, involving the removal of predictors in which only one instance differed from the others (i.e., all zeros and only one “1” value); these were considered as near-zero variance predictors.

### Training and Test Set

The MS^2^ data set contains multiple spectra of the same chemical or stereoisomers; therefore, it was necessary to avoid including the same chemicals in the training and test set, as this would lead to a positive bias in model performances. The division of the training and test set was done based on the first 14 characters of the corresponding InChIKey, as this reflects the bond connectivity and avoids having stereoisomers in both test and training sets.^47^ The data sets were slightly imbalanced, with more instances without an alert than with an alert (Table 1); therefore, the createDataPartition() function from the caret package was used to create balanced splits of the data for the training (70%) and test set (30%) to have a similar proportion of substances with the alert in the training and test set. The data set for the organophosphorus structural alert was highly imbalanced, with only 7 unique substances with an alert (corresponding to 47 spectra) in a test set of 582 substances in total (corresponding to 2198 spectra). Training models on extremely imbalanced data sets is challenging and likely to hamper the prediction accuracy. Hence, 628 additional MS^2^ spectra of 40 substances with an organophosphorus alert retrieved from NIST23^48^ were included, thereby reducing the imbalanced nature of the data set (Table 1). These additional ESI HRMS NIST23 spectra were collected by using the same filter criteria as described above for the MassBank spectra. For both the training and test sets, more spectra were present than unique substances, indicating that multiple spectra were present for the same chemical. Nevertheless, no further spectra were removed to avoid a loss of data.

**Table 1:** Specifications of the Different Training and Test Sets used for Model Training Per Structural Alert Group

		training set		test set	
structural alert	data type	total	with alert	total	with alert
aromatic amine	spectra	8697	3362	3485	1256
	chemicals	1447	291	619	124
organophosphorus	spectra	6044	595	2326	175
	chemicals	1379	36	590	15

### Model Training

**Figure 1:**
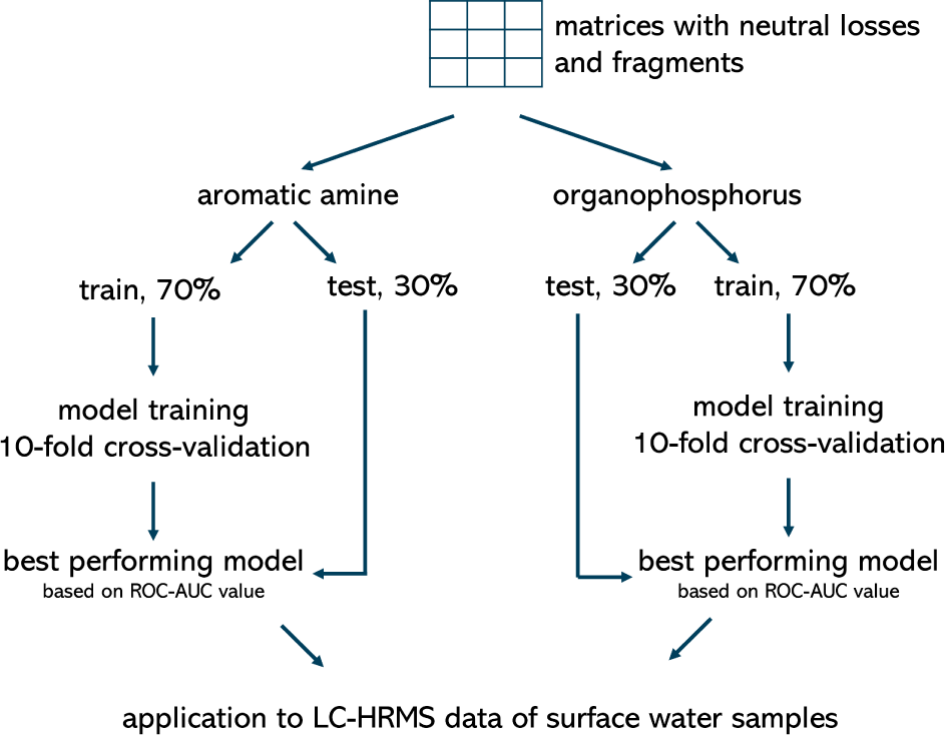
Schematic overview of the different steps taken for the generation of the training and test set, validation, and application to surface water samples.

Model training was run on multiple cores using the parallel^38^ and doParallel^49^ packages. At first, four machine learning algorithms were implemented for model training: a random forest classifier (rf), a single-layered feed-forward neural network (nnet), extreme gradient boosting (xgbTree), and a radial-kernel support vector machine (svmRadial). These algorithms were chosen because of their suitability for binary classification problems, their successful application for mass spectrometry data^50^ and similar tasks,^19,21,51^ and their accessibility in the caret package. The area under the receiver operating characteristic curve (AUC-ROC) was used as an optimization metric in the model training, which is suitable for binary classification purposes. The models were trained using 10-fold cross-validation. The performance of the different models was assessed using the AUC-ROC values, allowing us to take into account the true positive rate and false positive rate. The true positive rate and false positive rate were considered the most important metrics here, as the number of true positives should be as high as possible, whereas the number of false positives should be preferably as low as possible. Figure 1 gives a schematic overview of model development. Recursive feature elimination, using the *rfe()* function from the caret package with 10-fold cross-validation, was applied to the best-performing models to potentially enhance performance and robustness as well as reduce model complexity. To reduce computing time, it was decided to perform recursive feature elimination on the top 25% most important variables only while discarding the bottom 75%.

### Application to Samples

Samples spiked with compounds containing aromatic amines and organophosphorus groups were used to evaluate the performance of the trained models. More specifically, the aromatic amine model was applied to samples (ultrapure water, drinking water, and surface water) spiked with 27 aromatic amine-containing chemicals at a final concentration of 1 μg/L (Table S5). These samples had earlier been analyzed using the LC-HRMS method described in Been et al.^17^ Using the patRoon workflow described below, MS^2^ data for 25 out of 27 compounds could be retrieved. The organophosphorus model was applied to six spiked QC samples and four dust samples from the study by Belova et al.,^52^ which contained 8 organophosphorus-containing chemicals at a final concentration of 0.1 ng/μL (Table S6). Using the patRoon workflow described below, MS^2^ data for 7 of the 8 compounds could be retrieved. Furthermore, to evaluate the models on actual samples, reversed-phase liquid chromatography (RPLC)-HRMS NTS data of three surface water samples from the river Rhine and three surface water samples from the river Meuse collected in The Netherlands during a previous study were used.^17^ In this previous study, the samples were analyzed using an Orbitrap Fusion Tribrid mass spectrometer (Thermo Fisher Scientific) with electrospray ionization. The full scan ranged from 80 to 1300 *m*/*z* with a resolution of 120,000 fwhm. MS^2^ spectra were recorded using data-dependent acquisition with higher-energy collisional dissociation in the stepped collision energy mode. The raw RPLC-HRMS files, acquired in positive ionization mode, were converted into the open-source format .mzML using the msconvert tool of ProteoWizard.^53^ They were analyzed using patRoon^54^ (v 2.3.3), and features were obtained and grouped using the OpenMS^55^ algorithm (v 3.0.0). After grouping, peak qualities were calculated, and feature groups with a Gaussian Similarity score below 0.3 were removed. Basic filtering was applied for a retention time between 2.7 and 27 min and a minimum intensity of 10,000. Feature groups that were not present in two of the three replicates were removed, and feature groups present in the blank were removed as well if their intensity was <10 x blank intensity. The best-performing models were applied to the obtained MS^2^ spectra of the feature groups. Formula candidates and fingerprints were generated using SIRIUS^20^ (v 5.8.2), CSI:FingerID^26^ and GenForm.^56^ Potential compounds were annotated in patRoon using MassBank release version 2024.06.^57^ The NTA Study Reporting Tool (SRT) was used in the preparation of this manuscript.^58,59^

## Results and Discussion

### Structural Alerts

Many of the 187 structural alerts in ToxAlerts that were selected for this study had similar structures or contained similar SMARTS patterns. Similar structural alerts were grouped to reduce the number of classes that needed to be assessed and thereby improve performance. In total, 32 structural alert groups were assigned (Table S1), some of which are present in well-known potentially hazardous chemicals, e.g., organophosphorus being common in pesticides^60^ or flame retardants^61^ and carbamates in pesticides.^60^ Other structural alert groups are more general, such as epoxides, azo groups, and aliphatic halides.

The success of training classification models depends on the availability of training data, here, MS^2^ spectra. The number of substances and relevant MS^2^ spectra in MassBank with a structural alert varied largely between different structural alert groups, as can be seen in Table S1 and Figure S1. In the MassBank data set, 99 of the 187 individual alerts were found, and some substances contained multiple alerts, resulting in 375 unique combinations of structural alerts. Here, we focused on ESI positive ionization mode only; therefore, the number of substances with the structural alert available in the MassBank data set also depends on the chemical properties of the substances with the alert. If a specific alert is hardly present in substances that have been measured and deposited in MassBank, then fewer spectra will be available. Furthermore, measurement bias affects the availability of training data: more fragmentation spectra are available for (groups of) substances that have been more intensively studied. Any MS^2^ database, including MassBank, is a biased data set as it contains known substances of interest to (environmental) chemists. Therefore, it is a biased reflection of the chemical space measurable with LC-HRMS and further.^4,62^

The structural alert group with the largest number of unique substances (*n* = 415) and relevant MS^2^ spectra (*n* = 4582) in MassBank was the aromatic amine group. Aromatic amines are commonly used in the industrial synthesis of dyes, rubber, and drugs^63^ and are subsequently released into the environment via industrial effluent.^64,65^ They have been detected in surface waters and groundwater, among others.^65^ While aromatic amines are a very broad group of structural alerts, the organophosphorus group is smaller, with 142 relevant MS^2^ spectra of 16 unique substances, but has a more specific structure. Organophosphorus pesticides like methyl parathion, parathion, isocarbophos, and quinalphos have been detected in surface waters as well.^66^ Based on these considerations, the most abundant aromatic amine structural alert and the more specific organophosphorus structural alert were used as a case study to investigate the possibility of predicting structural alerts directly from the MS^2^ spectra.

### Curating Tandem Mass Spectra

The fragments and neutral losses were rounded to 1 decimal to yield binned data with a bin size of 0.1 *m*/*z*. Spectral binning reduces the number of variables; thereby, the resulting models become more robust toward alignment errors.^28^ After preprocessing, where only duplicate, empty (i.e., all instances equal to 0), and near-zero variance predictors were removed, the number of predictors varied per alert and data type. For the organophosphorus alert, 1661 unique fragments and 2098 unique neutral losses were used for model training, while for the aromatic amine alert, there were 1754 fragments and 2119 neutral losses. The fact that more bins of neutral losses compared to bins of fragments are computed could potentially be due to neutral losses having two sources of variability, namely the *m*/*z* of the fragments and the *m*/*z* of the precursor. On the other hand, with fragments, the only source of variability is the *m*/*z* of the fragment itself. MS^2^ spectra of different instruments (orbitrap and quadrupole time-of-flight, QTOF) were combined to obtain a data set of sufficient size for training purposes. Although Orbitrap and QTOF MS^2^ spectra are comparable within specific collision energy ranges,^67,68^ it is possible that training separate models for each type of instrument could lead to higher performance, as these are expected to be more robust against deviating collision energies. However, this approach would require sufficient training data for each specific mass analyzer. Moreover, the primary goal of our work was to develop a universal approach that can accommodate data generated by different instruments, ensuring broad applicability and enabling a wide range of uses for this method for prioritization purposes.

### Model Training and Interpretation

The distribution of other structural alerts for the random sample of spectra for the training and test set, without the alert of interest, was representative of the full data set (Figure S1). As a result, we deemed the use of a stratified sampling strategy unnecessary. The organophosphorus data set is imbalanced (9.8% spectra with alert), whereas the aromatic amines data set is less imbalanced (38.7% spectra with alert), mainly caused by the high occurrence of this structural alert (Table 1).

**Figure 2:**
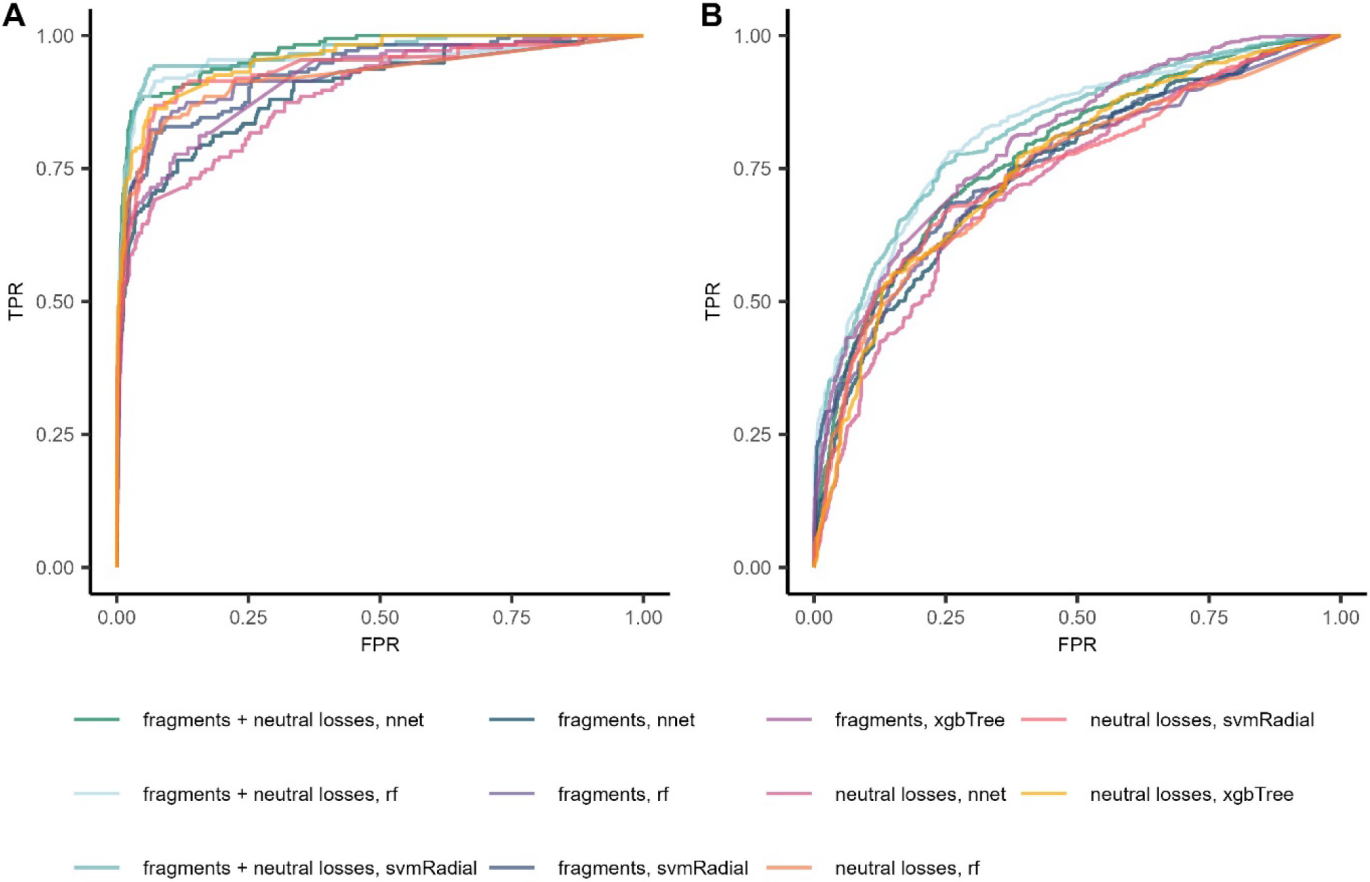
ROC curves on the test set data of the developed structural alert models with (A) organophosphorus and (B) an aromatic amine. The *x*-axis shows the false positive rate (FPR) and the *y*-axis shows the true positive rate (TPR). The following abbreviations were used: rf (random forest), nnet (single-layered feed-forward neural network), xgbTree (extreme gradient boosting) and svmRadial (radial-kernel support vector machine).

The performance of machine learning models can be assessed and optimized by various metrics, and the selection depends on the purpose of the model.^69^ Here, performance was evaluated following a precautionary principle; i.e., the risk of missing a potentially toxic feature should be kept as low as possible. In terms of prioritizing toxic features, this translates into maximizing the true positive rate (recall or sensitivity) to reduce the risk of misclassifying potentially toxic features as not containing an alert. On the other hand, false positives (i.e., features incorrectly prioritized as containing an alert) were considered less problematic from a risk management perspective; however, the fraction of such features should be kept as low as possible to reduce manual interrogation of the nontoxic features and the increased workload associated with this. As a result, the AUC-ROC was deemed a suitable metric for the purpose of NTS and was used to select the optimum classification model (Figure 2).

The best-performing organophosphorus model was a neural network using the combination of neutral losses and fragments as input variables, with a size of 1 and a decay of 0.1. On the test set, the model yielded an AUC-ROC value of 0.97 and classified 114 spectra out of 175 correctly as “organophosphorus alert present” (TPR of 0.65), while exhibiting a false positive rate of 0.01 (Table S3). For aromatic amines, the best model uses the combination of fragments and neutral losses as input variables and is built with a random forest algorithm, with 500 trees and an “mtry” value of 87. On the test set, the model has an AUC-ROC value of 0.82 and classifies 723 spectra out of 1256 correctly as “aromatic amine alert present”, exhibiting a false positive rate of 0.14 (Table S4). The challenges in the model training for the aromatic amines can, among others, be explained by the lack of diagnostic fragments.^70^ Although we found a diagnostic neutral loss (17.02655 *m*/*z*, potentially corresponding to the loss of NH_3_) in our previous study based on *in silico* MS^2^ data,^29^ the model training results show that the best-performing model is based on the combination of fragments and neutral losses. Moreover, the bin size of 0.1 *m*/*z* might affect the diagnostic power of this neutral loss because other less relevant neutral losses might fall into the same bin. Although smaller bin sizes, e.g., 0.01 or 0.001 *m*/*z*, might increase the diagnostic power of some fragments and neutral losses, they will lead to a tremendous increase in variables. Moreover, it is expected that more MS^2^ data will lead to increased performance. The promising performances obtained show that the trained models can be included in NTS workflows to prioritize features with structural alerts.

### Variable Importance

Interpretation of the trained machine learning models increases the trustworthiness of the models, and it enables the discovery of new patterns in the data; therefore, we investigated which neutral losses and fragments exhibited high importance in the trained models. The top 25 most important variables from the best-performing model were compared with group-specific fragments and neutral losses found in the literature, in particular from studies focusing on organophosphorus pesticides^71−74^ (Figure S5). For example, the fragment at 327.1 *m*/*z* was found to be the second most important feature and corresponded to a characteristic triphenyl phosphate ion (C_18_H_16_O_4_P^+^) fragment with an exact mass of 327.07807 Da previously reported by Hu et al.^73^ Furthermore, the fragment at 265.0 *m*/*z* might correspond to another characteristic fragment of C_13_H_14_O_4_P^+^ although its exact mass slightly deviates (i.e., 265.06242 Da, which would result in the bin at 265.1 *m*/*z*). The same goes for the characteristic fragment of C_13_H_12_O_3_P^+^ with an exact mass of 247.05186 Da, which deviates slightly from the 10th most important variable 247.0 *m*/*z*. These discrepancies between the fragment masses could be caused by the lower resolution used when MS^2^ spectra are acquired with HRMS instruments, especially with Orbitrap instruments. Regarding aromatic amines, the diagnostic neutral loss (17.02655 *m*/*z*) found in our previous study^29^ was absent in the top 25 most important variables. A comparison of the most important fragments and neutral losses (Figure S6) with existing literature is challenging because, to the best of our knowledge, such characteristic electrospray ionization MS^2^ fragments or neutral losses have yet to be reported. Therefore, findings from this study can serve as a starting point for mechanistic investigations aimed at discovering diagnostic aromatic amines fragments.

### Application of Both Models to Experimental NTS Data

Of the 25 aromatic amine-containing compounds with MS^2^ data in the spiked samples, 23 were successfully flagged by the developed model, with probabilities ranging from 0.651 to 1 (Table S5). For the organophosphorus model, 4 out of 7 spiked compounds with MS^2^ data were flagged, with probabilities ranging from 0.993 to 0.998 (Table S6). Two of the compounds flagged, namely, triphenyl phosphate and tricresyl phosphate, were also positively classified in actual (unspiked) dust samples from the study of Belova et al. (0.998 and 0.997 probability, respectively). The 3 compounds that were not flagged by the model, namely, 2-ethylhexyl diphenyl phosphate, resorcinol bis(diphenylphosphate), and bisphenol A bis(diphenylphosphate), formed sodium adducts [M + Na]^+^, which the model was not trained on, possibly explaining why they were not flagged. During data processing of the LC-HRMS data of the surface water samples, 45,647 features were extracted and yielded, after alignment and grouping, 8,161 feature groups. Further retention time, peak quality, and intensity (>10,000 counts) filtering alongside blank subtraction and componentization reduced the number of LC-HRMS features to 386, of which 352 had an MS^2^. Fragments and neutral losses were calculated for these features using MS^2^ spectra.

Seven of the feature groups yielded a probability score >0.5 for the class “organophosphorus alert present”, indicating that only a few features containing this substructure are present in the considered data set. Assigned formulas, highest-scoring candidates, and CSI:FingerID scores on the four bits of the CSI:FingerID fingerprint representing phosphate and other oxygen–phosphorus bonds are shown in Table S7. For six features, one or more of the top three predicted formulas included a phosphorus element. However, no candidate structures could be matched in MassBank, except for one feature group (M397_R1098_6504), which showed a match (0.88) with fluopyram—a fungicide that does not have an organophosphorus group but was also prioritized using the aromatic amines model (see below). The inability to find MassBank matches for the other flagged features underscores a challenge already encountered during model training: the limited availability of MS^2^ data for compounds with this specific structural alert. Nevertheless, these findings suggest the potential presence of unknown features characterized by an organophosphorus structural alert in the surface water samples, which should be further investigated to tentatively elucidate their structures. Regarding the “aromatic amine” alert, 194 feature groups yielded a probability score >0.5 and were further investigated. Assigned formulas, highest-scoring candidates, and CSI:FingerID scores on the two bits of the CSI:FingerID fingerprint representing a primary aromatic amine and secondary aromatic amine are shown in Table S7. For 11 of these feature groups, potential candidates were found in MassBank, of which nine contained an aromatic amine, whereas the other two contained a tertiary amine group. It is likely that these compounds might yield similar fragment ions and neutral losses to substances with an aromatic amine structural alert. Nevertheless, this application shows that it is possible to apply the developed models to nontarget screening data of environmental surface water samples.

### Research Significance and Future Directions

The approach developed here for predicting the presence of structural alerts can be utilized in environmental, exposomics, and human (bio)monitoring studies. It can rapidly highlight potentially significant features that require further investigation, either by applying other *in silico* tools or through additional experimental work like targeted analysis. Findings from this study show that based on raw and unfiltered MS^2^ spectra, it is possible to predict whether detected features potentially contain specific structural alerts associated with toxic effects, without the need to first predict molecular fingerprints from the data. The developed models can be used to both prioritize features in suspect and nontarget screening, as well as for more specialized applications such as (high-throughput) effect-directed analysis (EDA).^75^ In experimental toxicity testing with EDA or bioassays, a challenge is to associate the observed effect in the bioassay with a relevant feature(s), as each tested fraction still contains multiple features. Application of the developed models can help in narrowing the number of features potentially involved in the observed effects. For prioritization purposes, the proposed approach can be used to rapidly screen through a large number of acquired MS^2^ spectra to highlight the features with structural alerts and focus further identification efforts on these features. Furthermore, given that it is complementary to the calculation of molecular fingerprints from MS^2^ spectra, e.g., by SIRIUS,^20^ the proposed approach can be combined with recently developed tools such as MS2Tox^18^ or MLinvitroTox^19^ to further reduce the number of features for which toxicity/activity predictions need to be computed.

Results obtained here indicate that structural alerts can be predicted from the MS^2^ spectra. However, this is not necessarily the case for all structural alerts, as not all alerts can necessarily be linked to specific fragments, neutral losses, or combinations in MS^2^ data (e.g., halogens and epoxides). In the future, multiclassifier models could be trained to detect the presence of more structural alert groups, provided enough data is present to train performing models. Additionally, future algorithmic developments might improve the performance further. In particular, the evaluation of additional classification algorithms and more extensive feature engineering could help improve performance. Data preprocessing could be optimized by adjusting the bin size and using different strategies for variable selection. However, selecting the most suitable bin size is complex; higher resolutions result in a larger number of variables, increase computation time, model complexity, and require more training data. Additionally, this could lead to more alignment errors, as fragments or neutral losses may be split between different bins due to mass errors.^28^ Larger bin sizes and thus lower resolution overcome these problems but will lead to information loss. These disadvantages of uniform binning upon mass error could potentially be avoided by using Gaussian binning, which has been applied to NMR spectroscopy data^76^ but is still to be implemented on HRMS data. Future research can explore other variables that are acquired along the MS^2^ during data acquisition, e.g., signal intensity. Moreover, ongoing advancements in the field of metabolomics can serve as a foundation, as developed strategies can be equally applicable to the environmental analysis of small molecules.

This study showed that it is possible to build classification models on experimental fragmentation spectra acquired with positive electrospray ionization. We were able to apply the developed models to the NTS data of surface water samples and prioritize a set of features that potentially contain the aromatic amine structural alert. Both models can aid in pinpointing chemicals that are potentially hazardous to the environment and prioritize them for identification efforts. The possibility of predicting structural information related to the hazard of the molecule, without the use of fingerprints, is a valuable insight and can be used as a stepping stone for further research into the prioritization of NTS features in environmental samples. This approach could find applications in various nontarget screening studies of environmental samples. Overall, here we showed the potential of obtaining information on the potential hazard of an NTS feature based on the raw experimental MS^2^ data.

## Data Availability

The NIST-23 license agreement prohibits including spectra from it; we therefore cannot share the organophosphorus models and the full training and test set for this structural alert class. A subset of the training and test set, including MassBank spectra only, the aromatic amine model, and R code is shared on GitHub (https://github.com/KWR-Water/StructuralAlerts).

## Supporting Information Available

Overview of structural alert groups, model performances, and prioritized features (XLSX)
Model performance data and NTA SRT (PDF)
