# Supplementary material for: Machine Learning-based Classification for the Prioritization of Potentially Hazardous Chemicals with Structural Alerts in Nontarget Screening
Source: Environ Sci Technol. 2025 Mar 7;59(10):5056–65. doi: 10.1021/acs.est.4c10498 (PMC11924234; doi:10.1021/acs.est.4c10498)
Supplement: Supplementary file 2 — es4c10498_si_002.pdf [file es4c10498_si_002.pdf]

Machine learning-based classification for the prioritization of potentially hazardous chemicals with structural alerts in non-target screening

SUPPLEMENTARY INFORMATION

Nienke Meekel*[a,b], Anneli Kruve[c,d], Marja H. Lamoree[b], Frederic M. Been[a,b]

[a] KWR Water Research Institute, P.O. Box 1072, 3430 BB Nieuwegein, The Netherlands

[b] Chemistry for Environment and Health, Amsterdam Institute for Life and Environment (A-LIFE), Vrije Universiteit De Boelelaan 1085, 1081 HV Amsterdam, The Netherlands

[c] Department of Materials and Environmental Chemistry, Stockholm University, SE-106 91 Stockholm, Sweden

[d] Department of Environmental Science, Stockholm University, SE-106 91 Stockholm, Sweden

* corresponding author: nienke.meekel@kwrwater.nl

Summary: 10 pages, 6 figures, 2 tables, NTA study reporting tool.

Figure S1. Distribution of unique substances in total dataset, and 'no alert' class of training and test set S2
Figure S2. Precision-recall curves for the organophosphorus models, obtained on the test set data. S3
Figure S3. Precision-recall curves for the aromatic amine models, obtained on the test set data. S4
Figure S4. Results of the recursive feature elimination for aromatic amine model. S5
Figure S5. Top 25 most important variables for the best performing organophosphorus model. S6
Figure S6. Top 25 most important variables for the best performing aromatic amine model. S7
Table S8. Overview of R script and data files. S8
Table S9. Overview of code chunks S9
NTA study reporting tool S10

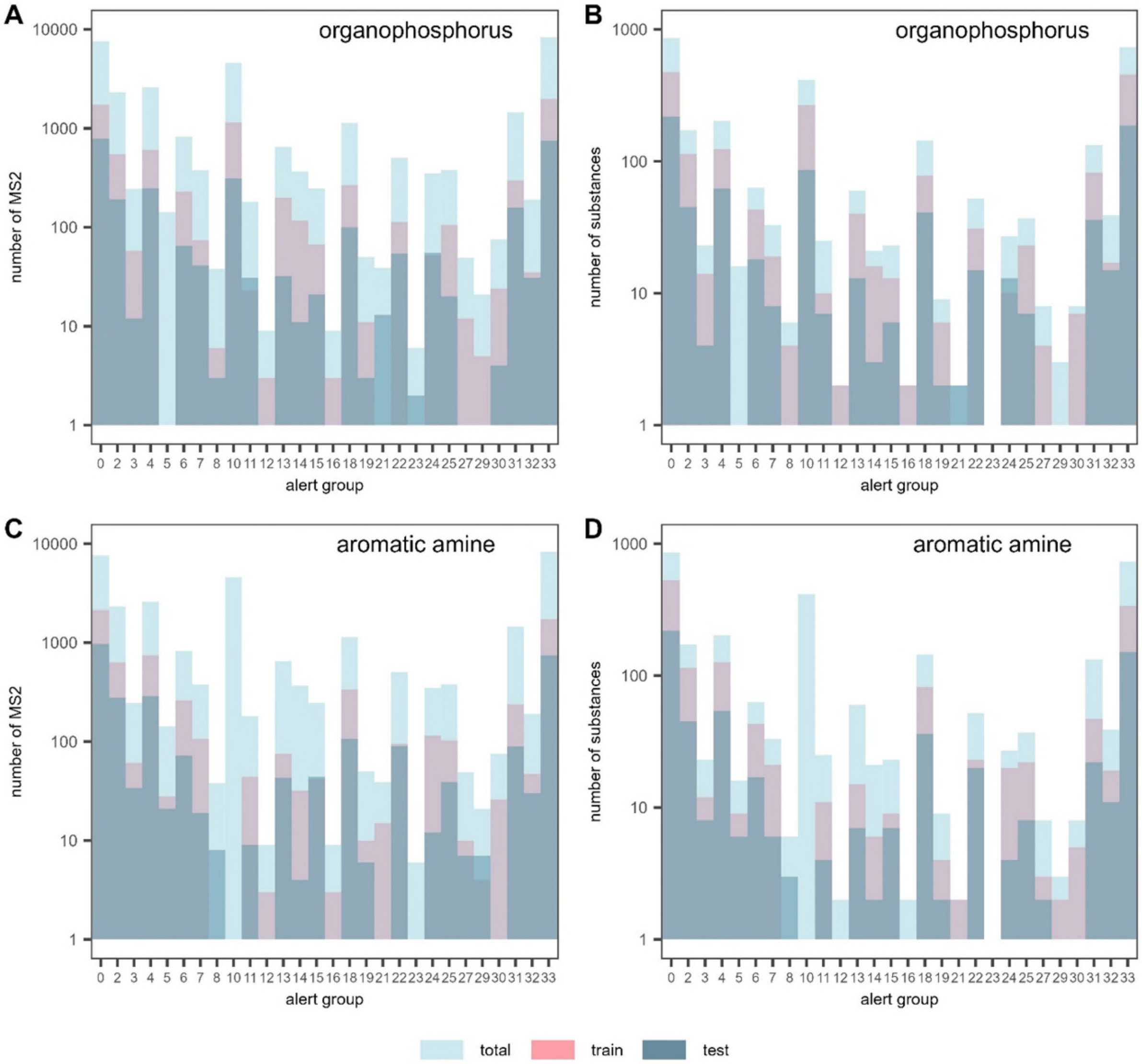

Figure S1 – Distribution of unique substances and MS^2 spectra across structural alert groups in the dataset, focusing on both the total dataset and the subset lacking a specific structural alert group, divided into training and test sets.

- The 'total' bar represents the proportion of MS^2 spectra and substances with the alert in the entire dataset. Note that this value is not the sum of the 'train' and 'test' bars, as these bars only reflect the subset of MS^2 spectra and substances in the 'no alert' class within the training and test sets.

- Panels (A) and (C) display the distribution of MS^2 spectra across structural alert groups.

- Panels (B) and (D) show the distribution of unique substances across structural alert groups.

- Panels (A) and (B) focus on the class without the organophosphorus alert, while (C) and (D) focus on the class without the aromatic amine alert.

Structural alert group codes 1-32 correspond to the groups listed in Table S1. Alert group 33 includes ungrouped alerts from Table S2, and alert group 0 corresponds to 'no alert'.

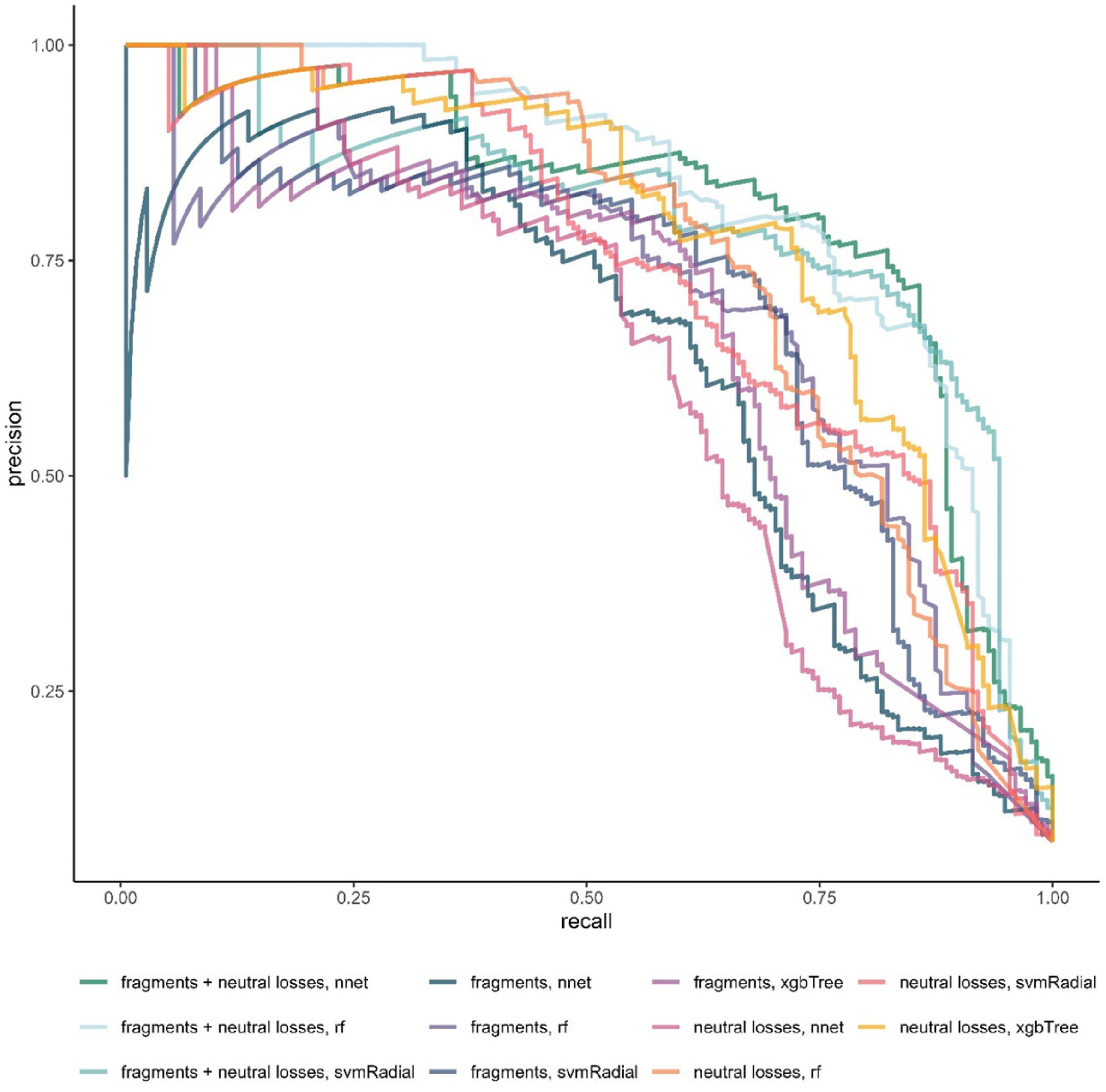

Figure S2 – Precision-recall curves for the organophosphorus models, obtained on the test set data.

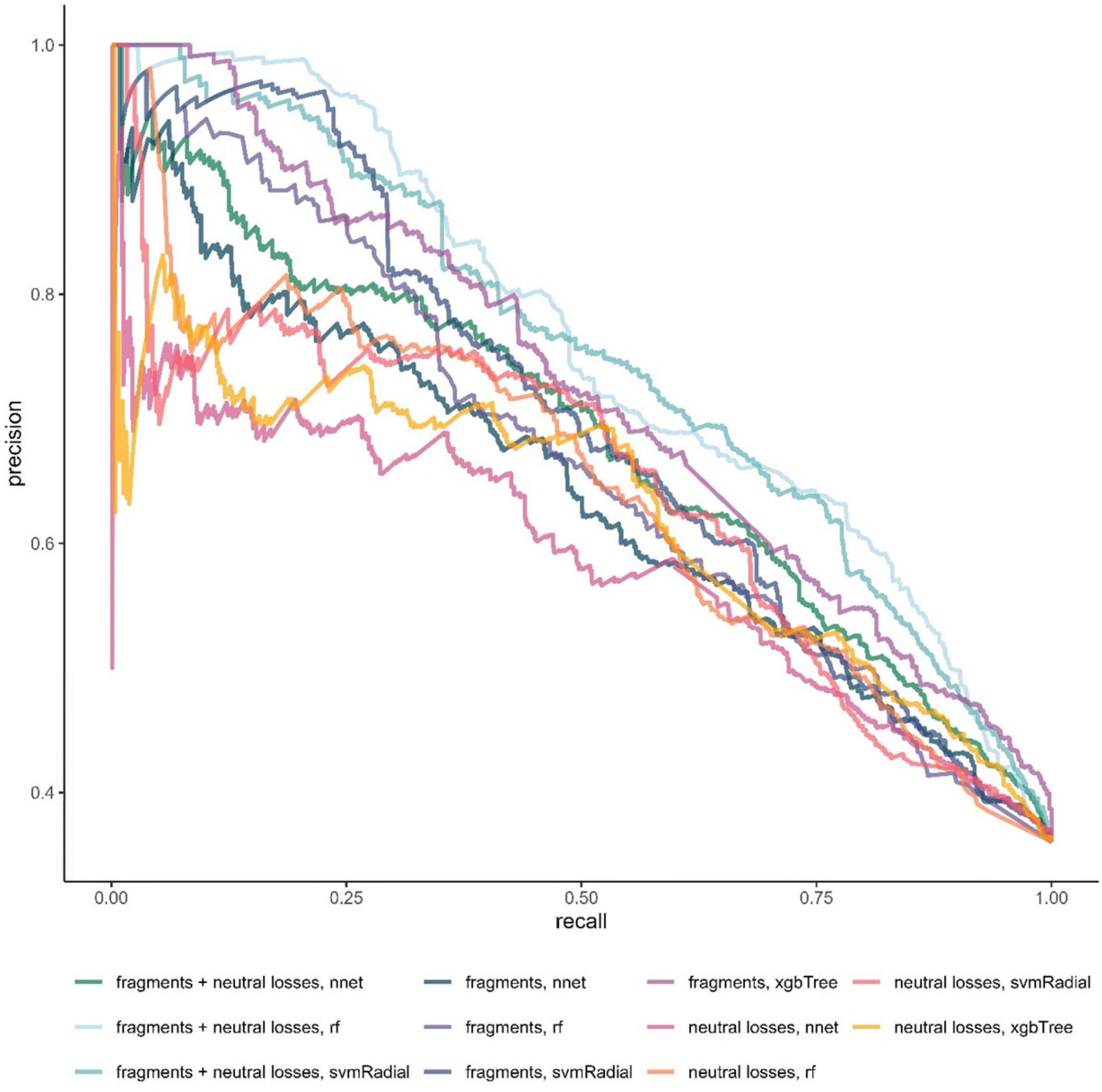

Figure S3 – Precision-recall curves for the aromatic amine models, obtained on the test set data.

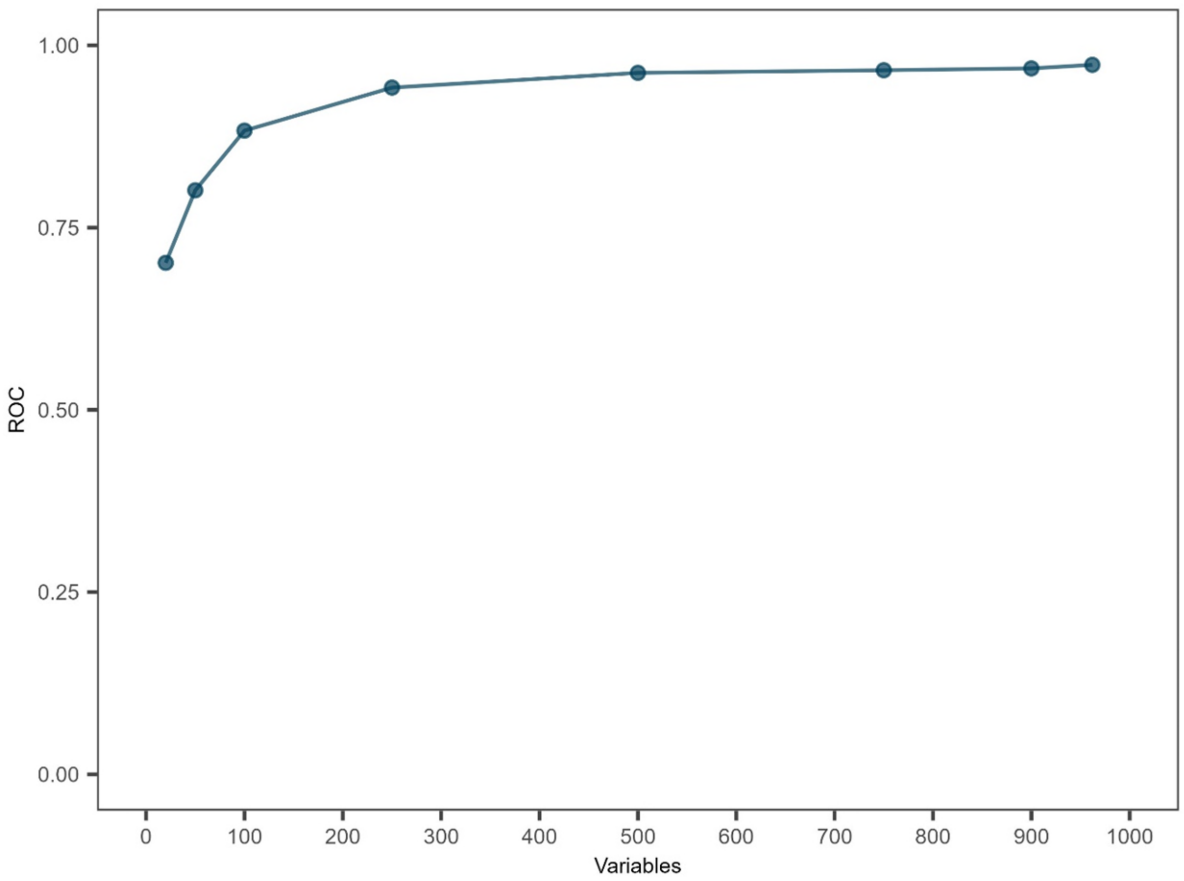

Figure S4 – Results of the recursive feature elimination on the top 25% most important variables for the best-performing model for aromatic amine, using 10-fold cross-validation with 5 repeats.

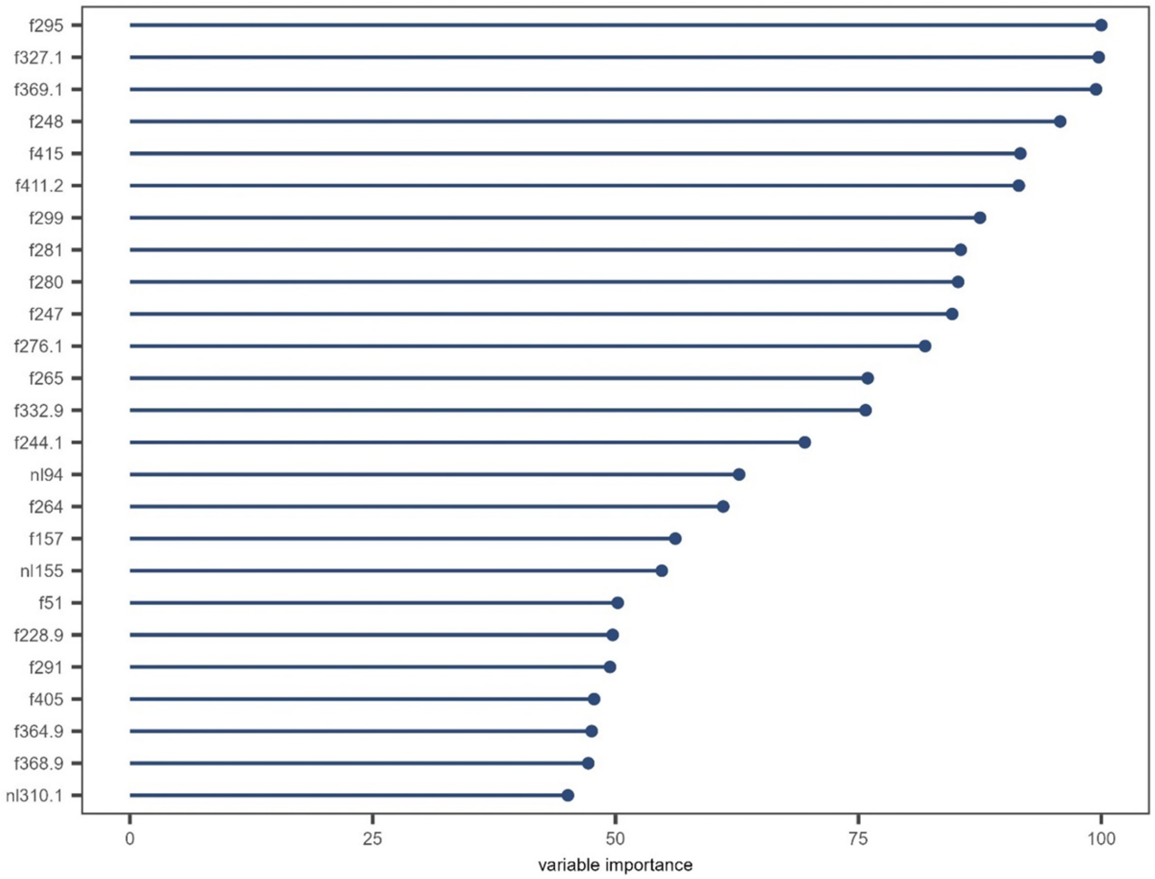

Figure S5 – Top 25 most important variables for the best performing organophosphorus model built with the single layer feed forward neural network algorithm. Fragments are indicated with ‘f’ and neutral losses are indicated with ‘nl’.

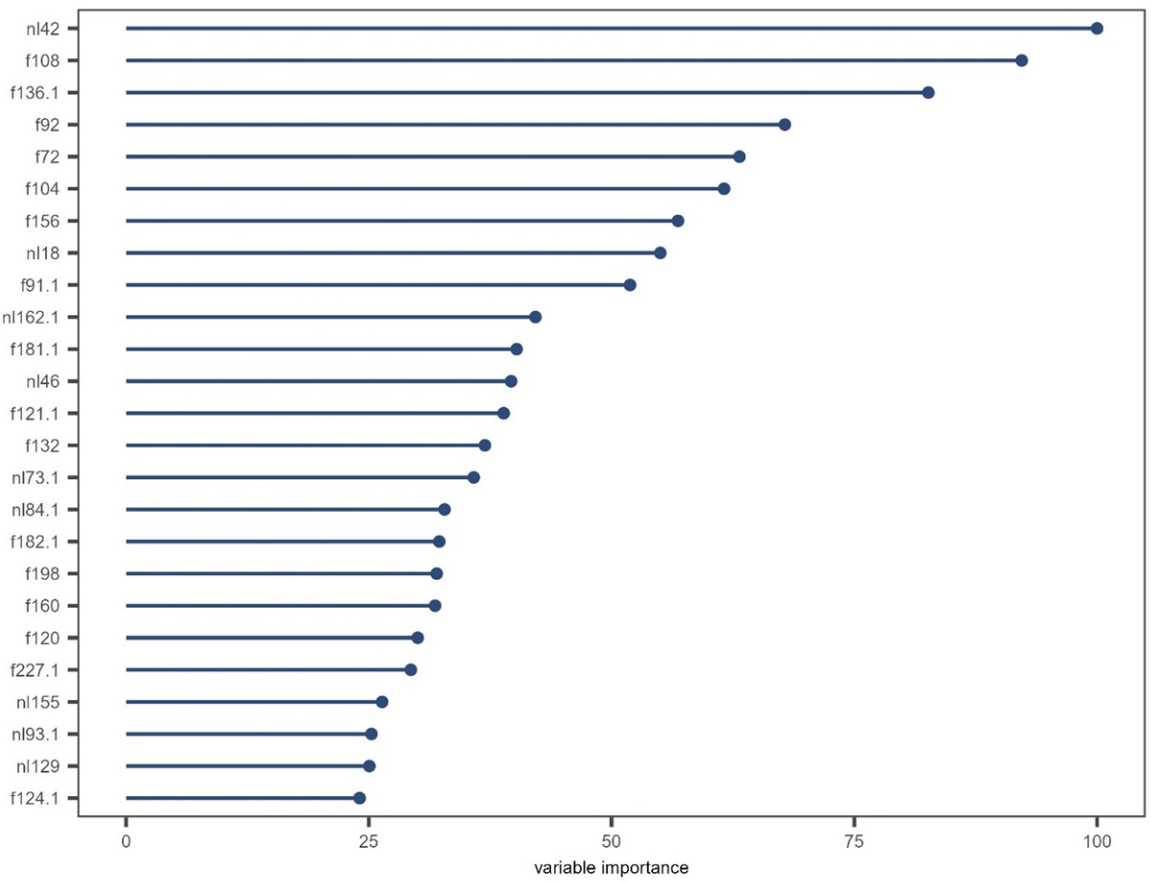

Figure S6 – Top 25 most important variables for the best performing aromatic amine model built with the random forest algorithm. Fragments are indicated with ‘f’ and neutral losses are indicated with ‘nl’.

Table S8 – Overview of R script and data files (AA = aromatic amine, OP = organophosphorus), available on https://github.com/KWR-Water/StructuralAlerts

name	type	description
data_test_aromaticamine_fragments.csv	dataset	test data for AA structural alerts, fragments
data_train_aromaticamine_fragments.csv	dataset	train data for AA structural alerts, fragments
data_test_aromaticamine_neutrallosses.csv	dataset	test data for AA structural alerts, neutral losses
data_train_aromaticamine_neutrallosses.csv	dataset	train data for AA structural alerts, neutral losses
data_test_aromaticamine_fragments_neutrallosses.csv	dataset	test data for AA structural alerts, fragments and neutral losses
data_train_aromaticamine_fragments_neutrallosses.csv	dataset	train data for AA structural alerts, fragments and neutral losses
data_test_organophosphorus_fragments_massbank_only.csv	dataset	test data for OP structural alerts, fragments (only MassBankEU spectra)
data_train_organophosphorus_fragments_massbank_only.csv	dataset	train data for OP structural alerts, fragments (only MassBankEU spectra)
data_test_organophosphorus_neutrallosses_massbank_only.csv	dataset	test data for OP structural alerts, neutral losses (only MassBankEU spectra)
data_train_organophosphorus_neutrallosses_massbank_only.csv	dataset	train data for OP structural alerts, neutral losses (only MassBankEU spectra)
data_test_organophosphorus_fragments_neutrallosses_massbank_only.csv	dataset	test data for OP structural alerts, fragments and neutral losses (only MassBankEU spectra)
data_train_organophosphorus_fragments_neutrallosses_massbank_only.csv	dataset	train data for OP structural alerts, fragments and neutral losses (only MassBankEU spectra)
MassBankEU_202206_fragments_pos_hrms.csv	dataset	fragments data set obtained in SI_script_fragments_neutrallosses.html
MassBankEU_202206_neutrallosses_pos_hrms.csv	dataset	neutral losses data set obtained in SI_script_fragments_neutrallosses.html
MassBankEU_202206_metadata_subset.csv	dataset	overview of metadata of relevant MS^2 spectra
structuralalertFunctions.R	R-script	functions for data pre-processing and prediction of OP structural alert and AA structural alert
model_aromaticamine.RDS	model	final random forest model for the prediction of an AA structural alert

Table S9 – Overview of code chunks (AA = aromatic amine, OP = organophosphorus), available on https://github.com/KWR-Water/StructuralAlerts

name	description
SI_script_fragments_neutrallosses.html	R-code to format MassBankEU data into data sets suitable for machine learning, containing fragments and neutral losses.
SI_script_aromatic_amine_combination_train_test.html	R-code to generate training and test set for the classification of MS^2 with AA structural alert. Combination of fragments and neutral losses
SI_script_aromatic_amine_train_test.html	R-code to generate training and test set for the classification of MS^2 with AA structural alert. Neutral losses and fragments separately.
SI_script_organophosphorus_combination_train_test.html	R-code to generate training and test set for the classification of MS^2 with OP structural alert. Combination of fragments and neutral losses
SI_script_organophosphorus_train_test.html	R-code to generate training and test set for the classification of MS^2 with OP structural alert. Neutral losses and fragments separately.
SI_script_model_training.html	R-code to train machine learning models for the classification of MS^2 spectra with an OP or AA structural alert.
SI_script_application_models.html	R-code to apply structural alert models in non-target screening workflow using patRoon.

NTA Study Reporting Tool

Please read before using!

Purpose: This Tool was developed for use by NTA researchers and reviewers to assess the quality of NTA study reporting. The resulting scores reflect solely whether the reporting is sufficiently complete and transparent (based on current, best available understanding of key study aspects in the environmental, food, exposomics, and metabolomics NTA communities). The Tool is not intended for evaluating the quality of the study or resulting data. The primary goal of using the SRT is to ensure that researchers provide all information about the study in sufficient detail to understand method details, replicate the analyses, enable comparisons across studies, and evaluate how study design choices may impact results.

We also encourage two supplementary uses of the Tool: 1) to guide study design - by considering what should be reported, a researcher is inherently encouraged to incorporate the necessary aspects into their study design, and 2) as a portal to relevant reference content and resources, which are available at the BP4NTA website (www.nontargetedanalysis.org) and via active hyperlinks within the SRT.

Notes & Guidance: The "Example Information to Report" column provides a brief list of representative items relevant to each sub-category - not all are required or necessary for every study, especially given differences across studies in different fields (e.g., environmental vs. metabolomics) and varied study goals (e.g., toxicant discovery vs. biological analysis). Researchers and reviewers should use their expertise and discretion to determine which aspects pertain to a given study, and whether additional details beyond those explicitly listed are also critical to report. Additionally, certain sub-categories may not be relevant to a given study (hence the option to select "NA"), or may be less critical to the overall quality and completeness of reporting. To evaluate these aspects, we strongly encourage users to consider the study type and objectives (e.g., method development, performance evaluation, field application), as well as conceptual linkages across subcategories (e.g., between Statistical Analysis and Statistical Outputs). We also encourage reviewers to include a rationale, so that authors/researchers may readily address concerns. Please also note that the Sections (Methods and Results) are not intended to indicate the location in a manuscript where the information is reported - a user should consider the manuscript in its entirety (including any supporting documents and/or citations).

Scoring: *NA* = not applicable (gray); *3* (blue) is the highest score and *0* (red) is the lowest. See score explanation table for details (www.nontargetedanalysis.org/srt/#srt-score-table and summarized below).

✔ Toggle to show score colors vs. fillable fields

Section	Category	Sub-Category	Example Information to Report	Score (drop-down menu) NA 0 1 2 3	Rationale for score
Methods	Study Design	Objectives & Scope	• Study goals and hypotheses<br>• Scope of the study with respect to use of NTA / suspect screening<br>• Expected chemical and/or metabolite coverage of the approach and potential limitations	NA	Not relevant for this study, the suspect and non-target screening study itself was described in Béen et al. Water Research (2021): https://doi.org/10.1016/j.watres.2021.117612
		Sample Information & Preparation	• Sample collection/replication, handling/storage, preparation, extraction, & clean-up methods (and related QA practices)<br>• Intended use of samples (e.g., method development, compound identification, etc.)<br>• Development and intended use of blanks	NA	Not relevant for this study, the suspect and non-target screening study itself was described in Béen et al. Water Research (2021): https://doi.org/10.1016/j.watres.2021.117612
		QC Spikes & Samples	• Development of QC spikes/samples (e.g., isotopically labeled standards/spikes, native standard spikes, matrix pools)<br>• Intended use of QC spikes/samples (e.g., to monitor instrument performance, data normalization, evaluate signal response range, etc.) and associated calculations and/or visualizations (e.g., mass error of spiked QC compounds, plot of signal response over time, etc.)	NA	Not relevant for this study, the suspect and non-target screening study itself was described in Béen et al. Water Research (2021): https://doi.org/10.1016/ [illegible]
	Data Acquisition	Analytical Sequence	• Sample randomization and use of replicate injections (e.g., technical/analytical replicates, field/laboratory/biological replicates)<br>• Inclusion of blanks and QC samples in the acquisition sequence<br>• Information about single vs. multiple analytical batches	NA	Not relevant for this study, the suspect and non-target screening study itself was described in Béen et al. Water Research (2021): https://doi.org/10.1016/j.watres.2021.117612
		Chromatography	• Note: chromatography is intended to include any online separation technique used prior to mass spectrometric detection.<br>• Instrument specifications<br>• Method settings (e.g., column/guard, mobile phases, gradient, injection techniques)	NA	Not relevant for this study, the suspect and non-target screening study itself was described in Béen et al. Water Research (2021): https://doi.org/10.1016/j.watres.2021.117612
		Mass Spectrometry	• Instrument specifications<br>• Instrument calibration and/or tuning procedures<br>• Method settings (e.g., acquisition parameters, such as polarity, resolution, data-dependent vs. data-independent)	NA	Not relevant for this study, the suspect and non-target screening study itself was described in Béen et al. Water Research (2021): https://doi.org/10.1016/j.watres.2021.117612
	Data Processing & Analysis	Data Processing	• File conversion information (e.g., to open-source format, centroiding)<br>• Software program(s) used<br>• Workflow steps (e.g., peak picking, RT calibration, alignment, gap filling) and settings<br>• Feature detection thresholds (e.g., replicate detection criteria; min height, area, or S/N levels; comparison to occurrence/abundance in blanks)<br>• Data correction or normalization methods (e.g., peak area/height normalization or scaling, blank subtraction)	3	All steps are described in the materials & methods, and the R-script for data analysis is available in the supporting information as well.
		Statistical & Chemometric Analysis	• Software programs(s)/package(s) used & samples/sample groups to which analyses were applied<br>• Basic statistical analysis method goals (e.g., summarize data, evaluate variability, hypothesis testing, identify outliers), type (e.g., Wilcoxon rank sum test, Chi-square test, dispersion ratio evaluation, Spearman/Pearson evaluation), assumptions, and settings/thresholds<br>• Chemometric analysis method goals (e.g., prioritize features, compare/classify samples, evaluate relationships between features), type (e.g., differential analysis, principal component analysis, hierarchical clustering, dimensionality reduction, metabolomic pathway analysis), assumptions, and settings/thresholds	3	The feature prioritization based on structural alerts is described in the materials and methods, the application of the models is shown in the R-script which is available in the supporting information.
		Annotation & Identification	• Software program(s) used (or description of manual annotation/identification efforts)<br>• Libraries and databases used (including details such as chemical coverage, resolution, metadata inclusion; information about in-house databases)<br>• Workflow steps (e.g., formula assignment, suspect screening, MS/MS spectral interpretation or library matching)<br>• Workflow methods & settings (e.g., formula prediction method, scoring algorithms; mass error/RT tolerances, accepted match scores)	3	All steps are described in the materials & methods, and the R-script for data analysis is available in the supporting information as well.
Results	Data Outputs	Statistical & Chemometric Outputs	• Basic statistical outputs (e.g., adj. p-values, standard deviations, test statistics)<br>• Chemometric analysis results (e.g., reported classifications/groupings of features/samples, outlier removal, observed data trends, metabolomic pathway analysis)<br>• Visuals/plots (e.g., Venn diagrams, heatmaps, clustering dendrograms, volcano plots, box plots, network diagrams, PCA & loading plots, molecular network diagrams)<br>• New statistical metrics, algorithms, packages, and/or scripts	3	The new statistical scripts and algorithms are provided in the supporting information. The chemometric analysis results are shown in Table S5, with scores per feature group.
		Identification & Confidence Levels	• Reported identifications and associated confidence levels (e.g., levels described by Schymanski et al., *ES&T*, 2014)<br>• Supporting data for annotation/identification (e.g., formula match scores, fine isotope pattern, retention time match, MS/MS match scores, source of MS/MS spectra)<br>• For features with lower confidence IDs (i.e., not standard-confirmed), proposed tentative structures and other annotated data<br>• Semi-quantification or quantification data<br>• Exported MS/MS spectra (e.g., as a library, database, or deposition into online repository)	NA	Proposed tentative structures are reported in Table S5, no identification levels were given as this was not the purpose of this study.
	QA/QC Metrics	Data Acquisition QA/QC	• Quality: Adherence to QA/QC protocols for sample preparation and data acquisition<br>• Boundary: Description of the potential impacts of methods (sample prep, chromatographic, MS) on observable chemical space<br>• Accuracy: Reported chromatographic and mass accuracy<br>• Precision: Variability of observed retention time, precursor mass error, and abundance	NA	Not relevant for this study, the suspect and non-target screening study itself was described in Béen et al. Water Research (2021): https://doi.org/10.1016/j.watres.2021.117612
		Data Processing & Analysis QA/QC	• Quality: Outcomes of QC checks or filtering steps along the data processing & analysis workflow<br>• Boundary: Impact of data processing & subsequent analysis method(s) on observed chemical space, observed limits of detection/ID<br>• Accuracy: Performance measures (True Positive Rate, False Positive Rate, etc.) for known compounds or samples with known classification<br>• Precision: Reproducibility/repeatability of performance measures for known compounds or samples with known classification; Calculations such as False Discovery Rate, F1 score, etc.	NA	Not relevant for this study.

Scoring System Explanation

Score	Explanation
0	No elements of relevant reporting are present.
1	Some elements of relevant reporting are present, but major improvements are needed.
2	Most elements of relevant reporting are present, but minor improvements are needed.
3	All elements of relevant reporting are present.
NA	Reporting not relevant to the study.
